# Mutation Rates and Intrinsic Fidelity of Retroviral Reverse Transcriptases

**DOI:** 10.3390/v1031137

**Published:** 2009-12-04

**Authors:** Luis Menéndez-Arias

**Affiliations:** Centro de Biología Molecular “Severo Ochoa” [Consejo Superior de Investigaciones Científicas (CSIC) & Universidad Autónoma de Madrid], Campus de Cantoblanco, 28049 Madrid, Spain; E-Mail: lmenendez@cbm.uam.es; Tel.: +34 91 196 4494; Fax: +34 91 196 4420

**Keywords:** reverse transcriptase, fidelity, retroviruses, HIV, DNA polymerase, mutation

## Abstract

Retroviruses are RNA viruses that replicate through a DNA intermediate, in a process catalyzed by the viral reverse transcriptase (RT). Although cellular polymerases and host factors contribute to retroviral mutagenesis, the RT errors play a major role in retroviral mutation. RT mutations that affect the accuracy of the viral polymerase have been identified by *in vitro* analysis of the fidelity of DNA synthesis, by using enzymological (gel-based) and genetic assays (e.g., M13mp2 *lac*Z forward mutation assays). For several amino acid substitutions, these observations have been confirmed in cell culture using viral vectors. This review provides an update on studies leading to the identification of the major components of the fidelity center in retroviral RTs.

## 1. Introduction

Virulence, pathogenesis and the ability to develop effective antiretroviral drugs and vaccines are largely dependent on retroviral variation. Genetic diversity in retroviruses has been widely documented, particularly in the case of human immunodeficiency virus type 1 (HIV-1) and simian immunodeficiency virus (SIV) [1–3]. Genetic variation in HIV-1 has been estimated from nucleotide sequence analysis of sequential isolates taken from individuals infected with a known viral source. Mutation rates of 10^−3^ and 10^−4^ nucleotide substitutions per site per year have been obtained for the HIV-1 *env* and *gag* genes, respectively.

Several methods have been used to determine the mutation rates of retroviruses in tissue culture (*i.e.,* the mutation frequency in a single cycle of retroviral replication). These methods imply the use of a vector virus genome which contains a target gene for scoring mutations [for a review, see Ref. 4]. Examples of genes that have been used as mutation reporters are *lac*Z or its truncated peptide (*lac*Zα), the neomycin phosphotransferase gene (*neo*), the herpes thymidine kinase gene and the green fluorescent protein gene. The retroviral vector encoding the reporter gene is introduced into a packaging cell line and the virus produced is used to infect target cells lacking the *gag-pol-env* genes. The vector can complete one round of replication and integrate in the target cell genome to form a provirus. However, because the vector is unable to express any viral proteins, additional cycles of replication cannot occur. At this point, an appropriate selection of cultured cells can determine the wild-type or mutant phenotype of the target gene and mutant frequencies can be obtained.

Mutation rates based on the inactivation of the reporter gene have been determined for several retroviruses, such as spleen necrosis virus (SNV), Rous sarcoma virus (RSV), murine leukemia virus (MLV), bovine leukemia virus (BLV), HIV-1 and human T-cell leukemia virus I (HTLV-I), and range from 2 × 10^−5^ to 6 × 10^−6^ per nucleotide per replication cycle [5–16]. The large differences obtained could be attributed in part to the different vectors and packaging and target cells used in these experiments. For example, reported estimates of the mutation rate of MLV ranged from 2 × 10^−6^ [8] to 2 × 10^−5^ [7]. Nevertheless, these values are well above the values reported for DNA-based microbes which range from 10^−7^ to 10^−11^ [17; for a review, see Ref. 18].

Retroviruses are RNA viruses that replicate through a DNA intermediate. Retroviral polymerases [*i.e.,* reverse transcriptases (RTs)] are multifunctional enzymes possessing RNA- and DNA-dependent DNA polymerase, RNase H, strand transfer and strand displacement activities but devoid of 3′→5′ exonuclease activity [reviewed in Refs. 19,20]. The double-stranded DNA resulting from reverse transcription is integrated into the host cell genome, where it is stably maintained as a provirus. Cellular DNA polymerases are responsible for the replication of the integrated viral DNA. Cellular RNA polymerase II transcribes the proviral DNA into RNA genomes that are packaged into virions. Unlike cellular DNA polymerases, viral RTs are devoid of 3′→5′ exonucleolytic proofreading activity. Average base substitution error rates for proofreading-proficient DNA polymerases (*i.e.,* DNA polymerases δ, γ, and/or ε) are about 10^−6^ to 10^−7^ [21,22], and 10 – 100 times lower than those of retroviral RTs. Based on those studies, it has been assumed that retroviral variation is largely a result of errors made by RT, although cellular RNA polymerase II as well as viral and host factors do have an effect on the retroviral mutation rate. In this review, we will discuss on different mechanisms influencing retroviral mutation rate with an emphasis on recent developments towards understanding the relationship between RT structure and its fidelity of DNA synthesis.

## 2. Viral and Host Factors Influencing Retroviral Mutation Rate

**Figure 1.**
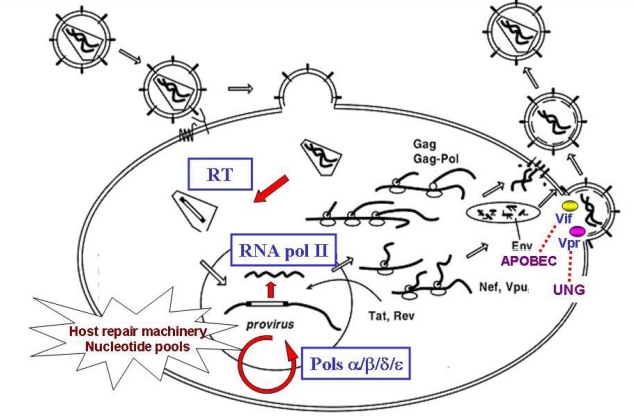
Major steps of the retroviral replication cycle and host factors that could influence the viral mutation rate.

The cellular transcriptional machinery, physiological fluctuations of dNTP pools and asymmetric error repair contribute to viral variation in the host cell (Figure 1). The contribution of the cellular RNA polymerase II is real but largely unknown. Early observations on the fidelity of wheat-germ RNA polymerase II *in vitro* that ranged from one error every 250 to one error in every 200,000 nucleotides polymerized, depending on the mutation type and on the sequence and structure of the template [23] suggested that RNA transcription was relatively error prone and contributed to retroviral mutagenesis.

Enzymatic studies carried out *in vitro* with DNA-dependent RNA polymerases revealed that misincorporation leads to slow addition of the next nucleotide, whereas a mismatched RNA 3′ end can be removed with factors that stimulate the polymerase cleavage activity (e.g.*,* Gre A in *E. coli*, or TFIIS in human cells) [24,25]. Recent reports have also demonstrated that transcriptional fidelity and proofreading do not require cleavage-stimulatory factors, but are intrinsic properties of RNA polymerases [26; reviewed in Ref. 27]. Sydrow *et al*. [28] have shown that RNA polymerase II evolved mismatch-specific fidelity mechanisms. Thus, certain DNA/RNA mismatches are efficiently formed, but impair RNA extension (e.g.*,* A:C, C:U, *etc.*), while others allow for RNA extension but are inefficiently formed and efficiently proofread by RNA cleavage (e.g.*,* G:G) [28]. X-ray analysis revealed that a T:U mismatch impairs RNA extension by forming a wobble base pair at the RNA polymerase II active site that dissociates the catalytic metal ion and misaligns the RNA 3′ end. On the other hand, an error rate as low as 1.9 × 10^−7^ to 2.3 × 10^−7^ mutations per copied nucleotide has been determined for the yellow fever virus RNA-dependent RNA polymerase [29], a value which is well above the generally accepted mutation rates for viral RNA polymerases of 10^−4^ to 10^−5^.

The relative contributions of viral RT and host cell RNA polymerase II to the high rate of HIV-1 mutation have been evaluated in a single cycle of virus replication, through the mutational analysis of HIV-1 long terminal repeats (LTRs). After sequencing 215 proviruses, O’Neil *et al.* found 21 independent mutations, 10 of them in both LTRs and 11 of them in only one of the LTRs [30]. In their experimental design, mutations found in both LTRs could have been introduced by either the RT or the RNA polymerase II, while the 11 mutations found in one of the LTRs could only have been introduced by the HIV-1 RT. These results provided direct evidence of the larger contribution of the RT to HIV-1 mutagenesis.

The RT is not the only viral protein that influences the retroviral mutation rate. Several retroviruses [e.g.*,* feline immunodeficiency virus (FIV), equine infectious anemia virus (EIAV), mouse mammary tumor virus (MMTV) and Mason-Pfizer monkey virus (MPMV)] encode a dUTP pyrophosphatase (dUTPase) [31,32]. Retroviral dUTPases reduce mutation levels by preventing the incorporation of uracil into the viral genome and therefore safeguarding efficient reverse transcription [33]. On the other hand, accessory proteins such as Vpr in HIV-1 have been shown to contribute to the accuracy of reverse transcription in single-cycle replication assays using the *lac*Zα gene as a mutational target. The mutation rate of a *vpr*^−^ vector mutant was about 4 times higher than that of the *vpr*^+^ parental vector (estimated at 3.4 × 10^−5^ per nucleotide and replication cycle), supporting the conclusion that the *vpr* gene partially accounts for the lower than predicted *in vivo* mutation rate of HIV-1 [11,34].

Vpr is a highly conserved regulatory protein of 96 residues that contains a central hydrophobic core domain with three α-helices surrounded by flexible N- and C-terminal domains [35]. Vpr is incorporated into virions and contributes both to reverse transcription and to the nuclear import of the HIV-1 preintegration complex [for recent reviews, see Refs. 36,37]. Vpr has been found to recruit the nuclear form of uracil DNA glycosylase (UNG2) into HIV-1 virions [38,39]. Replacement of Trp54 by Arg in Vpr is sufficient to disrupt its binding to UNG [39,40]. UNG is a key component of DNA repair mechanisms either in the nucleus or in the mitochondria, through the involvement of specific isoforms (UNG2 and UNG1, respectively) [41]. Although earlier reports indicated that Vpr-mediated incorporation of UNG2 into HIV-1 virions was required to modulate the virus mutation rate [39,42], recent reports suggest that UNG2 encapsidation has a detrimental effect on virus replication [43]. Although the later model proposes that Vpr induces proteasomal degradation of UNG2 in virus-producing cells, others have demonstrated that the Vpr-induced reduction of UNG2 levels in infected cells could be related to a negative transcriptional effect on UNG2 expression [44].

In addition, Vpr-independent packaging of UNG2 into HIV-1 virions has also been demonstrated [45]. This process appears to be mediated by the specific association between UNG2 and the integrase domain of the Gag-Pol precursor, and involves Leu172 in the viral integrase [46].

Apolipoprotein B mRNA-editing, catalytic polypeptide-like enzymes (APOBEC3) are cytidine deaminases that cause hypermutations of nascent retroviral genomes by deamination of cytidine residues [for recent reviews, see Refs. 47,48]. In the absence of the HIV-1 viral infectivity factor (Vif) protein, human APOBEC3G and APOBEC3F are encapsidated into budding virions. When these viral particles infect new target cells, two effects are observed: (i) the nascent viral reverse transcripts appear to be extensively mutated [49–51], and (ii) there is a significant reduction in the amount of viral DNA that accumulates in cells, due either to an inhibitory effect of APOBEC proteins on cDNA synthesis, or to an increase degradation of reverse transcripts [50,52–54; and references therein]. The proposal linking hypermutation to DNA degradation due to the UNG-mediated formation of abasic sites susceptible to degradation by cellular apurinic/apyrimidinic endonucleases [49,50] has been challenged by reports showing that various APOBEC proteins are able to inhibit MMTV or retrotransposons, with little or no detectable editing activity [55–57; reviewed in Ref. 58]. The virally encoded Vif protein allows HIV to overcome the activity of APOBEC proteins, resulting in a non-lethal level of G→A mutations. This proposal raises the intriguing possibility that these innate factors may directly shape the evolutionary diversity of retroviruses.

The contribution of other viral and cellular factors is still uncertain. The viral nucleocapsid protein (NC) could enhance the rate of viral DNA synthesis in regions of the template containing secondary structure [59], and biochemical studies have shown differences in the ability of the MLV RT to extend different mutated primers using RNA or DNA templates [60]. In addition, the nucleic acid chaperone activity of NC appears to play a key role in the selection of the 3′ polypurine tract (PPT) required for plus-strand DNA synthesis [61], and thereby safeguarding fidelity at this step of reverse transcription. In addition, cellular enzymes modifying nucleic acids (including host DNA repair enzymes) could potentially affect the retroviral mutation rate. Among them, the tumor suppressor protein p53 displays 3′→5′ exonuclease activity, and shows a preference for mispaired 3′ termini. In specific cell types, p53 could facilitate the extension of correctly paired 3′-terminus by HIV-1 RT [62,63].

## 3. Intrinsic Fidelity of Retroviral RTs: M13mp2 *lac*Z Forward Mutation Assays

The intrinsic fidelity of purified retroviral RTs can be analyzed *in vitro* by using enzymological (gel-based) or genetic assays [for an extensive review, see Ref. 64]. Gel-based assays include dNTP exclusion assays as well as determinations of kinetic constants (*i.e., k*_pol_ and *K*_d_ values, under pre-steady-state conditions) for the incorporation of correct or incorrect nucleotides on matched and mismatched template-primers. The dNTP exclusion assays are usually performed in the presence of three dNTPs, each one at a relatively high concentration, and provide a rough estimate of fidelity based on the observed primer extension efficiencies. In contrast, kinetic experiments (*i.e.,* misinsertion and mispair extension fidelity assays) provide detailed mechanistic insight on the processes governing the accuracy of DNA synthesis. However, these assays are time-consuming and usually restricted to the analysis of a small number of incorporation sites.

Genetic assays include the determination of the forward mutation rate of a reporter gene or the reversion of a nonsense codon. Forward mutation assays allow the exploration of a wide variety of sequence contexts that may help the identification of relevant hot spots related to reverse transcription errors. These assays provide information not only on single-nucleotide substitutions, but also on frameshifts and small deletions and insertions within the target gene. Silent mutations are not detected. However, forward mutation assays score for phenotypically detectable nucleotide changes at many different sites and in different sequence contexts, thereby providing a fidelity assessment based on a relatively large number of mutational target sites.

Mutation rates of purified RTs have been frequently determined with a forward mutation assay in which the *lac*Zα gene serves as a mutation reporter. In this assay, the substrate is a gapped double-stranded M13mp2 DNA duplex, where the *lac*Z sequence of one of the DNA strands has been deleted. Gap-filling reactions are carried out in the presence of RT and relatively high concentrations of all four dNTPs. Mutants are identified after transformation of appropriate bacteria by the white/blue color of M13 plaques revealed using X-Gal indicator plates [for a detailed description of the procedure, see Ref. 65]. Other reporter genes used in this type of assays are the HIV-1 *env* gene variable region 1 (V1) (cloned in M13) [66] or *lac*Z (cloned in vectors pBluescript or Litmus 29) [67,68]. Estimated error rates for retroviral RTs using forward mutation assays are given in Table 1.

Reported error rates for HIV-1 RT show a large variability that appears to originate in the gap-filling reaction, since differences are already detected while counting the number of mutant plaques. Another source of uncertainty is that the equivalence between mutant frequencies (white/pale blue *versus* blue plaques) and error rates is not straightforward, since detectable mutations and frameshifts do not occur with the same frequency at all sites, and sometimes, authors made their calculations based on partial sequences of the reporter genes. In addition, the observed variations could be explained in part by the different wild-type HIV-1 RTs used in these experiments [*i.e.,* homodimers (p66/p66) *versus* heterodimers (p66/p51), or RT variants derived from different viral strains, such NL4-3, HXB2, BH10 or NY5]. Forward mutation assays carried out under the same conditions revealed that HIV-1 RT was around 10 times less accurate than the AMV RT and 20 times less accurate than the Moloney MLV (Mo-MLV) RT [69,70,84]. In general, lentiviral RTs appear to be more error prone than oncoretroviral RTs. Thus, in M13mp2 *lac*Z forward mutation assays, feline leukemia virus (FeLV) RT was found to be approximately 11 times more faithful than feline immunodeficiency virus (FIV) RT [83].

**Figure 2.**
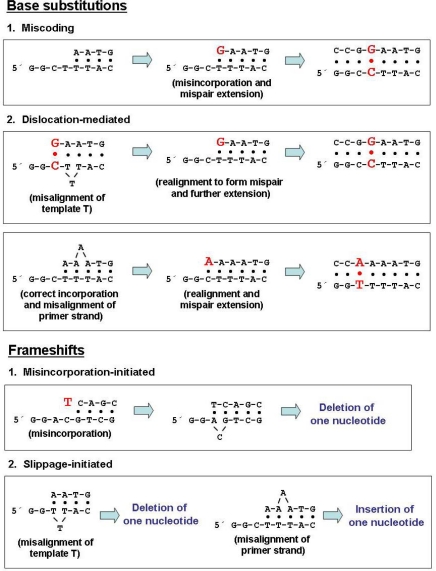
Proposed models and mutational intermediates leading to the generation of base substitutions and frameshift errors [adapted from Ref. 86].

SNV, MLV and HIV-1 RTs induce a similar broad spectrum of mutations during reverse transcription *in vivo* [6,11,85]. Most of them are substitution mutations. Transitions account for about 80% of the total number of mutations, whereas G→A transitions are the most frequent base substitutions. The comparison of the mutational spectra of HIV-1 and AMV RTs obtained *in vitro* using forward mutation assays revealed that HIV-1 RT shows a much higher error rate for frameshifts than the AMV RT (2.3 × 10^−4^ *versus* 1.0 × 10^−5^) [70,84]. Most of the frameshifts generated by HIV-1 RT appear at homopolymeric runs and could be explained by invoking a misalignment/slippage mechanism [70,86] (Figure 2).

Forward mutation assays carried out with RTs derived from the HIV-1 group M subtype B clade and from a phylogenetically distant group O isolate [87,88] demonstrated that the wild-type HIV-1 group O RT shows a very low frameshift fidelity in comparison with the subtype B enzyme [79]. During DNA synthesis, both highly divergent RTs are capable of generating substitution errors at specific sites at extraordinarily high rates (10^−2^ to 10^−3^) [69,70,79,81]. These hot spots are located at the boundaries of homopolymeric nucleotide runs. This location, together with the observed substitution specificity (mostly T→C and G→A mutations, derived from T:dGTP and G:dTTP mispairs) suggest that the hot spots result from a dislocation mechanism (Figure 2). This model assumes that the base substitution error is initiated at the end of a run by template-primer slippage. However, the formation of several mutational hot spots generated by HIV-1 RT when copying uracil-containing DNA templates could not be explained by dislocation mutagenesis, suggesting that direct misinsertion of the incorrect base is also an important contributor towards mutagenesis [80].

The comparison of mutational spectra of HIV-1 RT and other retroviral RTs revealed similar patterns for other closely-related lentiviral RTs, such as SIV_agm_ RT [81], whereas AMV RT and the prototype foamy virus (PFV) RT did not appear to make errors at specific spots [80,84]. PFV RT had similar error rates for single nucleotide substitutions, but made more deletions and insertions than the HIV-1 RT [80]. However, the analysis of mutations after a single round of reverse transcription using a replication-deficient vector system revealed that the replication of the foamy virus genome is more accurate than previously thought, while the proportion of deletions and insertions was almost negligible [89]. The discrepancies between the results obtained with PFV RT and the viral vector has been attributed in part to the viral Bet protein that could interact with members of the APOBEC family.

### Fidelity of retroviral RTs copying RNA *in vitro*

Forward mutation assays have been adapted for estimating the error rate of retroviral RTs while copying RNA *versus* DNA templates. The results obtained have been diverse, probably as a consequence of the different templates used in the DNA synthesis reaction. Using the M13mp2 *lac*Zα forward mutation assay, Boyer *et al.* [71] showed that the fidelity of HIV-1 RT was several-fold higher with RNA than with DNA. The largest differences were observed for single nucleotide substitutions and −1 frameshifts at homopolymeric runs suggesting that misaligned intermediates are formed or used less frequently with an RNA template-DNA primer than with a DNA template-DNA primer. Using a modified ΦX174 amber codon reversion assay, Hübner *et al*. [90] reported that the error rates for U:dGTP and rA:dGTP with HIV-1 RT were 20-fold and 7-fold higher, respectively, than the rates for the corresponding errors with a DNA template and the same sequence. In contrast, the base substitution fidelity of HIV-1 RT for errors likely to result from direct miscoding was found to be similar on RNA and DNA templates of the same sequence [66,67,71]. These results were consistent with the relatively small differences (about 2-fold) in mutation rates determined for RNA- and DNA-dependent DNA synthesis *in vivo* [91]. HIV-1 RT was also found to be less accurate than MLV RT [67] and AMV RT [71], demonstrating that error rates on RNA template are enzyme-dependent, as for DNA templates.

## 4. Assessing Fidelity of DNA Synthesis Using Nucleotide Incorporation Kinetics

Nucleotide selectivities based on the determination of kinetic parameters for the incorporation of correct and incorrect nucleotides on specific template-primers provide a valuable estimate of the fidelity of DNA synthesis [reviewed in Refs. 64,92,93]. In misinsertion fidelity assays, a binary complex is formed between the RT and the template-primer. Then, the ability to extend the primer in the presence of the correct or incorrect nucleotide is determined. The efficiency of nucleotide incorporation is measured by quantitative gel electrophoresis, and the data are analyzed to obtain kinetic parameters by using the Michaelis-Menten equation. The kinetic parameters *k*_cat_ and *K*_m_ can be determined under steady-state conditions. However, steady-state kinetic constants are not appropriate to obtain mechanistic insight into the DNA polymerization reaction, since the *k*_cat_ and *K*_m_ values are strongly influenced by the slow rate of dissociation of the template-primer from the enzyme (*k*_off_).

In contrast, pre-steady-state kinetic parameters obtained under conditions where the DNA concentration is in slight excess relative to the RT concentration provide a much better assessment of the nucleotide incorporation rate and the dNTP binding affinity. Rapid transient kinetics allow the determination of kinetics of nucleotide incorporation after time intervals ranging from 3 ms to several seconds, and therefore the measurement of the equilibrium dissociation constant (*K*_d_) for the interaction of dNTP and the RT/template-primer complex, as well as the reaction turnover (*k*_pol_). Nucleotide selectivity (e.g.*,* misinsertion ratio) can be estimated from the ratio between the *k*_pol_/*K*_d_ values for incorporation of incorrect and correct base pairs.

For retroviral RTs (e.g.*,* HIV-1 RT, MLV RT or SIV RT), the misinsertion fidelity of DNA-directed DNA synthesis is determined by a 10- to 100-fold reduction in the affinity for non-complementary dNTPs, and a 10- to 10,000-fold reduction in the rate of conformational change that limits the rate of nucleotide addition [94,95]. Reported misinsertion ratios are within the range of 10^−3^ to 10^−6^ and are lower for purine:purine and pyrimidine:pyrimidine base pairs [76,78,79,82,95–98].

The events leading to the fixation of a mutation involve nucleotide misincorporation, followed by extension of a mispaired template-primer. Efficient extension of mismatched 3′ termini is a major determinant of the low fidelity of HIV-1 and other retroviral RTs [99]. Extension of mispaired primer termini proceed at a rate one to three orders of magnitude faster than the dissociation of the retroviral RT from DNA, providing a mechanism for fixation of misincorporated nucleotides [95]. Mispair extension assays involve the determination of kinetic constants (*k*_pol_ and *K*_d_) for the incorporation of a correct nucleotide, using template-primers with matched or mismatched 3′ termini. Reported mispair extension efficiencies for MLV and HIV-1 RTs are usually within the range of 10^−2^ to 10^−4^. The highest mispair extension efficiencies are usually obtained for G:T or T:G mispairs [78,79]. Studies with RNA-DNA and DNA-DNA template-primers, bearing the same nucleotide sequences (except for the presence of U instead of T in the RNA template), showed that copying fidelity was around 10–25 times more accurate with the RNA template [96]. However, the number of sequence contexts analyzed in those studies was relatively small.

Despite the limitations of the steady-state kinetics approach, it should be noted that reported misinsertion and mispair extension ratios obtained under steady-state conditions were broadly similar to those calculated from pre-steady-state kinetic measurements. Steady-state misinsertion and mispair extension efficiencies were usually higher for HIV-1 RT and other lentiviral RTs [e.g., HIV-2, SIV, EIAV, FIV, and bovine immunodeficiency virus (BIV) RTs] than for MLV, FeLV and AMV RTs, in agreement with the results obtained using forward mutation assays [83,100–104]. Bovine leukemia virus (BLV) RT showed higher misinsertion fidelity than HIV-1 RT but lower than that of MLV RT, although the patterns of mispair elongation by the BLV enzyme suggested that its fidelity was similar to that reported for HIV-1 RT [105]. The MMTV RT was about 2- to 4-fold more error prone than AMV RT in misinsertion assays and showed a higher ability to extend mismatched template-primers [106]. On the other hand, misinsertion and mispair extension ratios obtained with RTs from porcine endogenous retrovirus were roughly similar to those obtained with MLV RT [107].

The fidelity of yeast LTR retrotransposon Ty1 RT was found to be comparable to that of AMV RT in gel-based fidelity assays [108], in agreement with error rate estimates of 10^−5^ misincorporations per nucleotide reported for the retrotransposed group II intron of *Lactococcus lactis* L1.LtrB [109]. On the other hand, it has been demonstrated that the RT of the LTR retrotransposon Tf1 of *Schizosaccharomyces pombe* shows a strong tendency to add non-templated nucleotides to the 3′-end of the nascent DNA, particularly in the presence of Mn^2+^ [110]. Under these conditions, Tf1 RT showed marked infidelity, although in the presence of Mg^2+^, nucleotide misincorporation levels were reduced in comparison with those obtained with the HIV-1 RT [110].

## 5. Structural Determinants of HIV-1 RT Fidelity

The HIV-1 RT is an important target for antiretroviral therapy [reviewed in Refs. 111,112]. During the last twenty years, many studies have been published describing the role of different amino acids in the acquisition of resistance to antiretroviral drugs, as well as the effects of mutations on nucleotide specificity.

The HIV-1 RT is a heterodimeric enzyme composed of two subunits of 66 and 51 kDa (p66 and p51, respectively), with subdomains termed ‘fingers’, ‘palm’, ‘thumb’ and ‘connection’ in both subunits and an RNase H domain in the larger subunit only [113,114]. As in other DNA polymerases fingers, palm and thumb subdomains of p66 form a large nucleic acid-binding cleft that extends into the connection subdomain and the RNase H domain. The bottom of the cleft is formed by the palm subdomain, which harbors the catalytic residues (Asp110, Asp185 and Asp186) that coordinate with two divalent ions. Binding of Mg^2+^ in metal site A (catalytic site) is required to obtain an RT which is catalytically competent for DNA polymerization [115], while binding of Mg^2+^ in site B is required for coordination with the triphosphate moiety and facilitates pyrophosphate dissociation [116]. Several residues in the vicinity of the catalytic triad, such as Lys65, Arg72, Asp113, Ala114, Tyr115 and Gln151 are involved in interactions with the incoming dNTP, while Leu74, Pro157, Phe160, Tyr183 and Met184 could indirectly affect dNTP binding [114]. Mutational studies have shown that molecular determinants of nucleotide specificity and fidelity of DNA synthesis map within the HIV-1 RT p66 subunit, mostly in the vicinity of the dNTP binding site. These analyses include measuring the effects of amino acid substitutions in the accuracy of DNA synthesis by using the M13mp2 *lac*Zα forward mutation assay (Table 2), as well as detailed studies of the kinetics of nucleotide incorporation with different template-primers.

**Figure 3.**
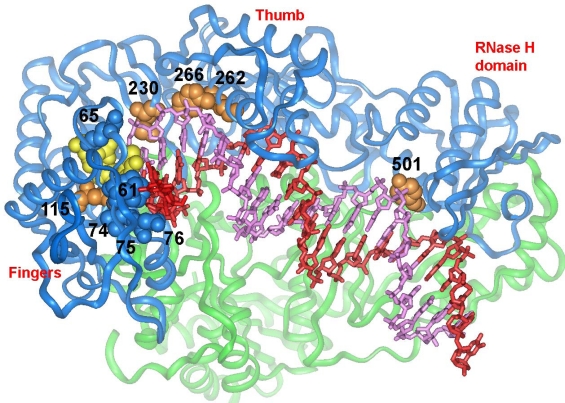
View of the nucleic acid binding cleft of HIV-1 RT showing the location of amino acids that influence fidelity of DNA synthesis (blue and orange CPK models). Ribbon diagrams are used for the representation of p66 (blue) and p51 (green). A stick representation is used for the template (red) and primer (magenta) strands of the DNA/DNA complex. The incoming dNTP is represented with a yellow CPK. Atomic coordinates were taken from Protein Data Bank file 1RTD [114].

Fidelity assays have demonstrated that the major structural determinants of the accuracy of DNA synthesis by HIV-1 RT are located in regions of the DNA polymerase domain, including: (i) dNTP binding site residues; (ii) residues that interact with the template strand; (iii) residues that interact with the primer strand; and (iv) minor groove binding track residues; as well as in the RNase H primer grip domain residues (Figure 3).

### 5.1. dNTP binding site residues

The side chains of Arg72 and Gln151 pack against the outer surface of the incoming dNTP, whereas the ribose ring of the nucleotide binds in a pocket defined by the side chains of Tyr115, Phe116 and Gln151. The side chain of Tyr-115 [as well as its equivalent residue in MLV RT (Phe155)] acts as a “steric gate”, preventing the incorporation of nucleotides with a 2′-hydroxyl group [128,129]. Although the substitution of Tyr115 by Phe renders a fully active RT [130,131], nonconservative substitutions at this position (e.g.*,* Y115A, Y115S, Y115V, *etc.*) rendered RTs with reduced misinsertion and mispair extension fidelity in nucleotide incorporation assays carried out under steady-state conditions [129,130,132]. Mutant Y115A showed a 4-fold lower fidelity than the wild-type RT in *lac*Z-based forward mutation assays [119] (Table 2). In contrast, Y115F and Y115V showed less than 2-fold differences in their error rates, in similar assays carried out with a deoxyuracil-containing DNA template [133]. Interestingly, the Y115A mutation increased by 2.3-fold the virus mutant frequencies *in vivo*, during one round of HIV-1 replication using the *lac*Z gene as a reporter gene [40]. A 2.8-fold increase in the mutation rate was also observed with a similar assay, when the F155W mutation was introduced in the RT-coding region of an MLV vector [134]. However, it should be noted that despite the strong evidence showing the role of Tyr115 in controlling fidelity of HIV-1 RT, nonconservative substitutions at this position have a deleterious effect on the specific DNA polymerase activity of the RT and render nonviable HIV-1 when introduced in an infectious clone [135].

Amino acid substitutions affecting neighboring residues Ala114 (*i.e.*, A114G, A114S) or Phe160 (*i.e.*, F160Y, F160W) did not seem to have a large impact in misinsertion and mispair extension fidelity [136,137]. However, the substitution of Asn for Gln151 rendered an RT with 13.1-fold decreased fidelity of DNA synthesis in M13mp2 *lac*Z forward mutation assays, in comparison with the wild-type enzyme [124]. In agreement with those observations, Q151N was found to decrease the viral mutation rate by 6-fold, in single-cycle replication assays [40]. Q151N RT was 120-fold less efficient at binding correct dNTP than wild-type RT [97]. However, mispair extension kinetics revealed that the higher accuracy of Q151N relates to its reduced ability to bind (*K*_d_) and chemically incorporate (*k*_pol_) nucleotide substrate onto mismatched template-primers [76]. It has been proposed that Gln151 could be important for tight binding of incorrect dNTPs, thereby contributing to the low fidelity nature of HIV-1 RT. Nevertheless, other amino acid substitutions at this position such as Q151M do not seem to affect HIV-1 RT fidelity in a significant manner [123,138].

Met184 of HIV-1 RT is an important residue for the acquisition of resistance to antiretroviral drugs such as lamivudine and emtricitabine [reviewed in Ref. 111]. Drug resistance mutations such as M184V or M184I are relatively common in heavily-treated patients. Several studies have shown that M184V confers increased misinsertion and mispair extension fidelity in comparison with the wild-type RT [139–142]. However, this mutation had a relatively small impact on the mutant frequency, as determined in forward mutation assays [73,119]. In contrast, M184I produced a 4-fold increase in the RT’s accuracy [74] (Table 2). The M184I RT variant showed a reduction in frameshift mutations when compared with the wild-type enzyme, as well as an increase in the number of mutations occurring outside runs of nucleotides [74].

Lys65 and Arg72 stabilize dNTP binding through interactions with the γ- and α-phosphates of the incoming dNTP. The substitution of Arg for Lys65 emerges under antiretroviral treatment, particularly with tenofovir [for a review, see Ref. 111]. M13mp2 *lac*Z forward mutation assays showed that the K65R mutation confers 8-fold higher accuracy than the wild-type enzyme [117], a result that is consistent with the 3-fold reduction in the mutant frequency observed in single-cycle replication assays [40]. The comparison of the mutational spectra obtained with K65R and wild-type RTs revealed increased frameshift fidelity in the case of K65R RT. In addition, the number of occurrences of a particular type of error at the same site (*i.e.,* hot spots) was reduced in reactions catalyzed by the mutant enzyme [117]. The substitution of Ala for Arg72 impairs polymerase activity by interfering with the pyrophosphate removal function of the RT [143,144]. Despite having similar fidelity in comparison with the wild-type enzyme, the mutant R72A RT was found to be highly error-prone for misincorporations opposite template T in the sequence context: 5′-CTGG, as demonstrated by kinetic experiments and forward mutation assays [118].

### 5.2. Residues that interact with the template strand

Several mutations in the fingers subdomain of the RT that involve residues interacting with the 5′ template overhang were shown to decrease the *in vitro* forward mutation frequency (Table 2). These amino acid substitutions include F61A [75], L74V [117,119], V75I [78,79], D76V [120], and R78A [121]. The largest effects on fidelity have been reported for mutant F61A. However, its mutational spectrum has not been reported. Phe61 together with Trp24 are required for accurate association of the HIV-1 RT with the template-primer [145]. Despite retaining significant DNA polymerase activity (as well as strand displacement activity) [75,146], Phe61 mutations were shown to cause a replication defect when introduced in viral clones [147]. In comparison with the wild-type RT, mutational spectra obtained with mutants L74V and V75I revealed only minor differences in the type of errors and their distribution across the *lac*Z reporter gene [79,117].

Glu89 is of particular interest because it interacts with the sugar-phosphate backbone of the template strand around template position −2, and appears to be important for RT activity and drug resistance. Despite the fact that the E89G RT displayed increased fidelity of dNTP insertion and mispair extension; forward mutation assays failed to demonstrate a significant effect of this mutation in fidelity [73,119] (Table 2). However, the mutational spectra of E89G and E89G/M184V RTs revealed a frameshift hot spot that was not found with the wild-type enzyme [73]. Mutant RTs having Ala, Asp, Asn, Gly, Lys, Ser, Thr or Val, instead of Glu89 showed reduced −1 frameshifting and increased +1 frameshifting suggesting that Glu89 can influence slippage of both strands [122]. An association between −1 frameshifting and reduced dNTP incorporation rates was also observed. A salt bridge between Glu89 and Lys154 appears to facilitate −1 frameshifting. This observation is consistent with the 7.5 to 10-fold reduction in −1 frameshifting observed for mutants K154A and K154R [122].

The effects of RT mutations L74V, D76V, R78A and E89G on the retroviral mutation rates have been analyzed in single-cycle replication assays [40]. The largest effects were observed with R78A and D76V that caused 3.5- and 2.5-fold reductions in the observed mutant frequencies in comparison with the wild-type HIV-1 vector. Interestingly, the combination of D76V and R78A produced a further significant reduction in the average mutant frequencies (0.151 ± 0.006 *versus* 0.015 ± 0.005 mutants per cycle for the wild-type and the double-mutant D76V/R78A, respectively).

### 5.3. Residues that interact with the primer strand

The largest effects on fidelity have been reported for mutations affecting the primer grip region of p66 (*i.e.,* residues 227–235 of the palm subdomain). RTs having Ala instead of Phe227, Trp229, Met230, Gly231 or Tyr232 were 40 to 76% less efficient than the wild-type enzyme in extending a mismatch in primer extension assays carried out with a mixture including all dNTPs [148]. On the other hand, single-cycle replication assays revealed that mutations F227A and W229A produced only a small decrease (<2-fold) in the mutant frequency, suggesting a relatively minor effect on fidelity [40]. However, we have previously shown that the primer grip mutation M230I increases the rate of T:G misinsertion by 16-fold in assays carried out under steady-state conditions [149]. M230I emerges as a compensatory mutation for the dNTP binding defect shown by an HIV-1 RT bearing Trp at position 115 [135]. A genetic screen based on the blue-white β-galactosidase complementation assay designed to detect G→A mutations arising during RNA-dependent DNA synthesis revealed that mutant RTs bearing mutations M230L or M230I were 20 to 70 times less faithful than the wild-type RT in the presence of low [dCTP]/[dTTP] ratios, but showed similar fidelity in assays carried out with equimolar concentrations of each nucleotide [150].

### 5.4. Minor groove binding track residues

DNA binding track interactions occur in the minor groove where the DNA undergoes a structural transition from A-like to B-form DNA [114,151]. Ile94, Gln258, Gly262, Trp266 and Gln269 are considered to be of potential importance for binding to duplex template-primer in this region. Alanine-scanning mutagenesis of residues 253–271 (α-helix H) and 277–287 (α-helix I) have shown that these structures have a role in template-primer binding and processivity. Amino acid substitutions involving α-helix H residues Gly262 and Trp266 were shown to increase the error rate of HIV-1 RT [125,126]. Mutant enzymes containing either G262A or W266A had decreased template affinity, processivity, and frameshift fidelity, and were able to synthesize a small amount of full-length DNA product, in comparison with the wild-type RT. Both mutations appear to be deleterious for viral replication [40].

### 5.5. RNase H primer grip domain residues

The RNase H primer grip domain was identified as a structural element of HIV-1 RT that is involved in the control of RNase H cleavage specificity [152]. In HIV-1 RT, the RNase H primer grip includes Tyr501, a residue that interacts with the DNA primer and facilitates its proper positioning in relation to the RNase H active site [153]. Tyr501 of HIV-1 RT is part of the DSXY conserved motif found in all retroviral RNase H domains, except RSV RT. The equivalent residue of MLV RT is Tyr586. The substitution of Phe for Tyr586 in MLV RT increased the *in vivo* mutation rate by approximately 5-fold [59]. DNA-sequencing analysis indicated that the Y586F mutation increased the frequency of base substitution mutations 17-fold within adenine-thymine tracts (AAAA, TTTT, or AATT sequences), which are known to induce bends in DNA. Another substitution at the equivalent position in HIV-1 (Y501W) produced a 2.7-fold increase in the mutant frequency obtained in one round of HIV-1 replication assays, using *lac*Z as a reporter gene [40]. In contrast, the RNase H primer mutation (I505A) had a non-significant effect on fidelity in this type of assays [40].

More recently, Mbisa *et al*. [154] showed that significant increases (2.1- to 3.8-fold) in the viral mutation rates could be obtained by introducing mutations S557A, A558V and Q559L in the RT-coding region of an MLV vector. These mutations as well as Y586F increased the frequency of deletions between the primer binding site (PBS) and sequences downstream of the PBS, and support a relevant role for the RNase H primer grip in controlling fidelity of DNA synthesis *in vivo*.

## 6. Biological Consequences of Increasing or Decreasing Fidelity: Questions and Perspectives

The publication of evidence suggesting that a mutation emerging under antiretroviral therapy (M184V) increased the fidelity of HIV replication [139] triggered hot debate on whether antiretroviral therapy could delay the appearance of drug-resistant viruses. Earlier clinical studies revealed that M184V had a minor impact in the evolutionary potential of the virus [155,156], although there is at least one report showing the delay in the development of resistance to amprenavir and efavirenz when M184V is present [157]. Nevertheless, *in vitro* studies suggest that the effects of M184V in fidelity are modest. Theoretical models considering the viral replication rate, fitness and fidelity of the virally encoded RT suggested that, in order for increases in fidelity to make an impact on the rate at which a subsequent variant appears, increases in polymerase fidelity should be very large [3,158]. Drug-resistant HIV-1 RTs such as M41L/T69SSS/L210W/R211K/L214F/T215Y and related enzymes, appear to show the largest increase in accuracy [77]. However, their effects on viral evolution have not been studied in detail.

On the other hand, an increase of the error rate above a critical threshold could lead to the loss of genetic information in a process termed “error catastrophe”. This strategy, termed lethal mutagenesis, is based on the concept that only a modest increase in the viral mutation rate is needed to render the virus non-viable [159–161]. The validity of lethal mutagenesis is supported by experimental evidence showing an inverse correlation between mutation rate and infectivity of several RNA viruses, including retroviruses [162–165]. Nucleoside analogues (e.g., 5-azacytidine, 5-hydroxydeoxycytidine, *etc.*) have been shown to effectively increase mutation rates of various retroviruses, including SNV, MLV, FIV and HIV-1 [159,166–169]. In most cases, these effects are the result of variations in intracellular dNTP pools that could affect virus titers, as well as the rate and spectrum of retroviral mutations [170]. It should be noted that variations in the dNTP pools may not affect all retroviruses in the same manner (e.g., MLV RT shows a higher affinity for dNTPs than HIV-1 RT [98]). Interestingly, certain drugs could alter dNTP pools and cause mutational bias. Thus, it has been reported that the administration of 2′-deoxycytidine and tetrahydrouridine can reverse the characteristic G→A mutational bias found in HIV-1 [171].

In summary, available data suggest that the control of the viral mutation rate could be a viable antiretroviral strategy. Still more work needs to be done in order to understand the molecular mechanisms involved in controlling fidelity not only at a molecular level (*i.e.*, intrinsic RT fidelity), but also related to the identification of intracellular factors that modulate the viral mutation rate (e.g., cellular polymerases and their regulators, and variations in the nucleotide pools). One of the defying future challenges in this area of research would be the design and development of mutagenic nucleoside analogues devoid of potential toxicity and carcinogenicity, and showing higher specificity for the retroviral RTs.

## Figures and Tables

**Table 1.** Estimated error rates of retroviral RTs, as determined by using forward mutation assays.

Enzyme	Vector and reporter gene	Template	Error rate	References
HIV-1 RT (group M subtype B)	M13mp2, lacZα	DNA	0.6 – 6.7 × 10^−4^	[69–79]
	pBluescript, *lacZ*	DNA	1.7 × 10^−4^	[67]
	pBluescript, *lacZ*	RNA	1.4 × 10^−4^	[67]
	M13, *env* V1	DNA	1.9 × 10^−4^	[66]
	M13, *env* V1	RNA	2.0 × 10^−4^	[66]
	Litmus 29 (uracil-containing DNA), *lacZ*	DNA	0.75 – 1.6 × 10^−4^	[68,80]
HIV-1 RT (group O)	M13mp2, *lacZ*α	DNA	5.5 × 10^−5^	[79]
SIV_agm_ RT	M13mp2, *lacZ*α	DNA	2.9 × 10^−5^	[81]
SIV_mne_ RT	M13mp2, *lacZ*α	DNA	1.2 × 10^−4^ (CL8) ^a^ 1.6 × 10^−5^ (170)	[82]
PFV RT	Litmus 29, *lacZ*	DNA	1.7 × 10^−4^	[80]
FIV RT	M13mp2, *lacZ*α	DNA	6.2 × 10^−5^	[83]
AMV RT	M13mp2, *lacZ*α	DNA	5.9 × 10^−5^	[69,84]
Mo-MLV RT	M13mp2, *lacZ*α	DNA	3.3 × 10^−5^	[69,84]
	pBluescript, *lacZ*	DNA	3.4 × 10^−5^	[67]
	pBluescript, *lacZ*	RNA	2.7 × 10^−5^	[67]
FeLV RT	M13mp2, *lacZ*α	DNA	5.8 × 10^−6^	[83]

a CL8 and 170 are strains of SIV_mne_ that infected the same pig-tailed macaque. The 170 strain is a representative clone of the late symptomatic phase of the infection.

**Table 2.** Accuracy of mutant HIV-1 RTs, as determined by using M13mp2 *lac*Zα forward mutation assays.

Mutation^a^	RT subunit composition^b^	Subdomain location	Mutant frequency (× 10^−4^)^c^	Fold-Change^d^	References
M41L/T69SAG/AMs	p66/p51^WT^ (HXB2)	fingers/palm	6.3 (97)	↓ **15.3**	[77]
M41L/T69SSG/AMs	p66/p51^WT^ (HXB2)	fingers/palm	5.9 (97)	↓ **16.3**	[77]
F61A	p66/p51^WT^ (HXB2)	fingers	8.3 (97)	↓ **11.7**	[75]
A62V/T69SAG/AMs	p66/p51^WT^ (HXB2)	fingers/palm	8.5 (97)	↓ **11.4**	[77]
A62V/T69SSG/AMs	p66/p51^WT^ (HXB2)	fingers/palm	19 (97)	↓ **5.0**	[77]
A62V/T69SSS/AMs	p66/p51^WT^ (HXB2)	fingers/palm	11 (97)	↓ **8.8**	[77]
K65R	p66/p66 (BH10)	fingers	10.6 (86)	↓ **8.1**	[117]
T69SAG	p66/p51^WT^ (HXB2)	fingers	20 (97)	↓ **4.8**	[77]
T69SSG	p66/p51^WT^ (HXB2)	fingers	12 (97)	↓ **7.5**	[77]
T69SSS	p66/p51^WT^ (HXB2)	fingers	24 (97)	↓ **4.0**	[77]
R72A	p66/p66 (HXB2)	fingers	340 (210)	↑ 1.6	[118]
L74V	p66/p66 (BH10)	fingers	50.5 (86)	↓ 1.7	[117]
	p66/p51^WT^ (BH10)		55 (192)	↓ **3.5**	[119]
V75A	p66/p51 (BH10)	fingers	281 (206)	↑ 1.4	[78]
V75F	p66/p51 (BH10)	fingers	112 (206)	↓ 1.8	[78]
V75I	p66/p51 (BH10)	fingers	69.6 (206)	↓ **3.0**	[78]
	p66/p51 (ESP49)		43.4 (83.1)	↓ 1.9	[79]
D76V	p66/p51 (BH10)	fingers	26 (232)	↓ **8.8**	[120]
R78A	p66/p51 (BH10)	fingers	28 (250)	↓ **8.9**	[121]
E89G	p66/p51^WT^ (HXB2)	fingers	62.6 (86)	↓ 1.4	[73]
	p66/p51^WT^ (BH10)		96 (192)	↓ 2.0	[119]
E89G/M184V	p66/p51^WT^ (HXB2)	fingers/palm	123 (86)	↑ 1.4	[73]
E89K	p66/p51^WT^ (HXB2)	fingers	77 (86)	↓ 1.2	[122]
E89S	p66/p51^WT^ (HXB2)	fingers	53 (86)	↓ 1.6	[122]
E89V	p66/p51^WT^ (HXB2)	fingers	64 (86)	↓ 1.3	[122]
Y115A	p66/p51^WT^ (BH10)	palm	763 (192)	↑ **4.0**	[119]
V148I	p66/p66 (HXB2)	palm	30 (261)	↓ **8.7**	[76]
	p66/p66 (SIV-CL8)		22 (178)	↓ **8.1**	[82]
Q151M	p66/p66 (BH10)	palm	55 (64)	↓ 1.2	[123]
Q151M^COMPLEX^	p66/p66 (BH10)	fingers/palm	31 (64)	↓ 2.1	[123]
Q151N	p66/p51 (BH10)	palm	20 (261)	↓ **13.1**	[124]
K154A	p66/p51 (BH10)	palm	125 (261)	↓ 2.1	[124]
Y183F	p66/p51^WT^ (BH10)	palm	303 (192)	↑ 1.6	[119]
M184I	p66/p51 (HXB2)	palm	24 (97)	↓ **4.0**	[74]
M184V	p66/p51 (HXB2)	palm	55.3 (86)	↓ 1.6	[73]
	p66/p51^WT^ (BH10)		228 (192)	↑ 1.2	[119]
D256A	p66/p66 (HXB2)	thumb	240 (200)	↑ 1.2	[125]
Q258A	p66/p66 (HXB2)	thumb	390 (200)	↑ 1.95	[125]
K259A	p66/p66 (HXB2)	thumb	300 (200)	↑ 1.5	[125]
L260A	p66/p66 (HXB2)	thumb	230 (200)	↑ 1.15	[125]
G262A	p66/p66 (HXB2)	thumb	860 (210)	↑ **4.1**	[126]
K263A	p66/p66 (HXB2)	thumb	290 (200)	↑ 1.45	[125]
W266A	p66/p66 (HXB2)	thumb	630 (210)	↑ **3.0**	[126]
Q269A	p66/p66 (HXB2)	thumb	510 (200)	↑ 2.55	[125]
R277A	p66/p66 (HXB2)	thumb	140 (160)	↓ 1.1	[127]
Q278A	p66/p66 (HXB2)	thumb	190 (160)	↑ 1.2	[127]
L279A	p66/p66 (HXB2)	thumb	150 (160)	↓ 1.1	[127]
C280A	p66/p66 (HXB2)	thumb	300 (160)	↑ 1.9	[127]
K281A	p66/p66 (HXB2)	thumb	140 (160)	↓ 1.1	[127]
L282A	p66/p66 (HXB2)	thumb	120 (160)	↓ 1.3	[127]
R284A	p66/p66 (HXB2)	thumb	170 (160)	↑ 1.1	[127]
G285A	p66/p66 (HXB2)	thumb	160 (160)	=	[127]
K287A	p66/p66 (HXB2)	thumb	120 (160)	↓ 1.3	[127]

a AMs stands for the additional mutations: L210W/R211K/L214F/T215Y; and Q151M^COMPLEX^ represents A62V/V75I/ F77L/F116Y/Q151M.

b RTs used in these experiments were homodimers (p66/p66) or heterodimers (p66/p51). The WT superscript indicates that the corresponding subunit contains a wild-type sequence. The reference viral strain is shown between parentheses. All of them are HIV-1 group M subtype B strains except for ESP49 that derives from an HIV-1 group O isolate and CL8 that is an SIV strain.

c The mutant frequencies indicated between parenthesis correspond to controls carried out with the corresponding wild-type RT.

d ↑ and ↓ stand for increased and decreased mutant frequency (inaccuracy), respectively.

## References

[1] Wei X, Ghosh SK, Taylor ME, Johnson VA, Emini EA, Deutsch P, Lifson JD, Bonhoeffer S, Nowak MA, Hahn BH, Saag MS, Shaw GM (1995). Viral dynamics in human immunodeficiency virus type 1 infection. Nature.

[2] Ho DD, Neumann AU, Perelson AS, Chen W, Leonard JM, Markowitz M (1995). Rapid turnover of plasma virions and CD4 lymphocytes in HIV-1 infection. Nature.

[3] Coffin JM (1995). HIV population dynamics *in vivo*: Implications for genetic variation, pathogenesis, and therapy. Science.

[4] Svarovskaia ES, Cheslock SR, Zhang W-H, Hu W-S, Pathak VK (2003). Retroviral mutation rates and reverse transcriptase fidelity. Front Biosci.

[5] Dougherty JP, Temin HM (1986). High mutation rate of a spleen necrosis virus-based retrovirus vector. Mol Cell Biol.

[6] Pathak VK, Temin HM (1990). Broad spectrum of *in vivo* forward mutations, hypermutations, and mutational hotspots in a retroviral shuttle vector after a single replication cycle: Deletions and deletions with insertions. Proc Natl Acad Sci USA.

[7] Monk RJ, Malik FG, Stokesberry D, Evans LH (1992). Direct determination of the point mutation rate of a murine retrovirus. J Virol.

[8] Varela-Echavarría A, Garvey N, Preston BD, Dougherty JP (1992). Comparison of Moloney murine leukemia virus mutation rate with the fidelity of its reverse transcriptase *in vitro*. J Biol Chem.

[9] Varela-Echavarría A, Prorock CM, Ron Y, Dougherty JP (1993). High rate of genetic rearrangement during replication of a Moloney murine leukemia virus-based vector. J Virol.

[10] Mansky LM, Temin HM (1994). Lower mutation rate of bovine leukemia virus relative to that of spleen necrosis virus. J Virol.

[11] Mansky LM, Temin HM (1995). Lower *in vivo* mutation rate of human immunodeficiency virus type 1 than that predicted from the fidelity of purified reverse transcriptase. J Virol.

[12] Parthasarathi S, Varela-Echevarría A, Ron Y, Preston BD, Dougherty JP (1995). Genetic rearrangements occurring during a single cycle of murine leukemia virus vector replication: characterization and implications. J Virol.

[13] Mansky L (1996). Forward mutation rate of human immunodeficiency virus type 1 in a T lymphoid cell line. AIDS Res. Hum. Retroviruses.

[14] Mansky LM (2000). *In vivo* analysis of human T-cell leukemia virus type 1 reverse transcription accuracy. J Virol.

[15] Huang KJ, Wooley DP (2005). A new cell-based assay for measuring the forward mutation rate of HIV-1. J Virol Methods.

[16] Laakso MM, Sutton RE (2006). Replicative fidelity of lentiviral vectors produced by transient infection. Virology.

[17] Drake JW (1991). A constant rate of spontaneous mutation in DNA-based microbes. Proc Natl Acad Sci USA.

[18] Sniegowski PD, Gerrish PJ, Johnson T, Shaver A (2000). The evolution of mutation rates: separating causes from consequences. BioEssays.

[19] Telesnitsky A, Goff SP, Coffin J, Hughes SH, Varmus H (1997). Reverse transcription and the generation of retroviral DNA. Retroviruses.

[20] Basavapathruni A, Anderson KS (2007). Reverse transcription of the HIV-1 pandemic. FASEB J.

[21] Kunkel TA, Alexander PS (1986). The base substitution fidelity of eucaryotic DNA polymerases. J. Biol. Chem..

[22] Matsuda T, Bebenek K, Masutani C, Hanaoka F, Kunkel TA (2000). Low fidelity DNA synthesis by human DNA polymerase-η. Nature.

[23] de Mercoryol L, Corda Y, Job C, Job D (1992). Accuracy of wheat-germ RNA polymerase II: General enzymatic properties and effect of template conformational transition from right-handed *B*-DNA to left-handed *Z*-DNA. Eur. J. Biochem.

[24] Erie DA, Hajiseyedjavadi O, Young MC, von Hippel PH (1993). Multiple RNA polymerase conformations and GreA: Control of the fidelity of transcription. Science.

[25] Thomas MJ, Platas AA, Hawley DK (1998). Transcriptional fidelity and proofreading by RNA polymerase II. Cell.

[26] Nesser NK, Peterson DO, Hawley DK (2006). RNA polymerase II subunit Rpb9 is important for transcriptional fidelity *in vivo*. Proc Natl Acad Sci USA.

[27] Saxowsky TT, Doetsch PW (2006). RNA polymerase encounters with DNA damage: Transcription-coupled repair or transcriptional mutagenesis. Chem Rev.

[28] Sydow JF, Brueckner F, Cheung ACM, Damsma GE, Dengl S, Lehmann E, Vassylyev D, Cramer P (2009). Structural basis of transcription: Mismatch-specific fidelity mechanisms and paused RNA II polymerase II with frayed RNA. Mol Cell.

[29] Pugachev KV, Guirakhoo F, Ocran SW, Mitchell F, Parsons M, Penal C, Guirakhoo S, Pougatcheva SO, Arroyo J, Trent DW, Monath TP (2004). High fidelity of yellow fever virus RNA polymerase. J Virol.

[30] O’Neil PK, Sun G, Yu H, Ron Y, Dougherty JP, Preston BD (2002). Mutational analysis of HIV-1 long terminal repeats to explore the relative contribution of reverse transcriptase and RNA polymerase II to viral mutagenesis. J Biol Chem.

[31] Elder JH, Lerner DL, Hasselkus-Light CS, Fontenot DJ, Hunter E, Luciw PA, Montelaro RC, Phillips TR (1992). Distinct subsets of retroviruses encode dUTPase. J. Virol..

[32] Köppe B, Menéndez-Arias L, Oroszlan S (1994). Expression and purification of the mouse mammary tumor virus *gag-pro* transframe protein p30 and characterization of its dUTPase activity. J Virol.

[33] Lerner DL, Wagaman PC, Phillips TR, Prospero-García O, Henriksen SJ, Fox HS, Bloom FE, Elder JH (1995). Increased mutation frequency of feline immunodeficiency virus lacking a functional deoxyuridine-triphosphatase. Proc Natl Acad Sci USA.

[34] Mansky LM (1996). The mutation rate of human immunodeficiency virus type 1 is influenced by the *vpr* gene. Virology.

[35] Morellet N, Bouaziz S, Petitjean P, Roques BP (2003). NMR structure of the HIV-1 regulatory protein Vpr. J Mol Biol.

[36] Zhao RY, Elder RT, Bukrinsky M (2007). Interactions of HIV-1 viral protein R with host cell proteins. Adv Pharmacol.

[37] Malim MH, Emerman M (2008). HIV-1 accessory proteins – Ensuring viral survival in a hostile environment. Cell Host Microbe.

[38] Selig L, Benichou S, Rogel ME, Wu LI, Wodicka MA, Sire J, Benarous R, Emerman M (1997). Uracil DNA glycosylase specifically interacts with Vpr of both human immunodeficiency virus type 1 and simian immunodeficiency virus of sooty mangabeys, but binding does not correlate with cell cycle arrest. J Virol.

[39] Mansky LM, Preveral S, Selig L, Benarous R, Benichou S (2000). The interaction of Vpr with uracil DNA glycosylase modulates the human immunodeficiency virus type 1 *in vivo* mutation rate. J Virol.

[40] Mansky LM, Le Rouzic E, Benichou S, Gajary LC (2003). Influence of reverse transcriptase variants, drugs, and Vpr on human immunodeficiency virus type 1 mutant frequencies. J Virol.

[41] Nilsen H, Otterlei M, Haug T, Solum K, Nagelhus TA, Skorpen F, Krokan HE (1997). Nuclear and mitochondrial uracil-DNA glycosylases are generated by alternative splicing and transcription from different positions in the UNG gene. Nucleic Acids Res.

[42] Chen R, Le Rouzic E, Kearney JA, Mansky LM, Benichou S (2004). Vpr-mediated incorporation of UNG2 into HIV-1 particles is required to modulate the virus mutation rate and for replication in macrophages. J Biol Chem.

[43] Schröfelbauer B, Yu Q, Zeitlin SG, Landau NR (2005). Human immunodeficiency virus type 1 Vpr induces the degradation of the UNG and SMUG uracil-DNA glycosylases. J Virol.

[44] Langevin C, Maidou-Peindara P, Aas PA, Jacquot G, Otterlei M, Slupphaug G, Benichou S (2009). Human immunodeficiency virus type 1 Vpr modulates cellular expresión of UNG2 via a negative transcriptional effect. J Virol.

[45] Willetts KE, Rey F, Agostini I, Navarro J-M, Baudat Y, Vigne R, Sire J (1999). DNA repair enzyme uracil DNA glycosylase is specifically incorporated into human immunodeficiency virus type 1 viral particles through a Vpr-independent mechanism. J Virol.

[46] Priet S, Navarro J-M, Gros N, Quérat G, Sire J (2003). Functional role of HIV-1 virion-associated uracil DNA glycosylase 2 in the correction of G:U mispairs to G:C pairs. J Biol Chem.

[47] Aguiar RS, Peterlin BM (2008). APOBEC3 proteins and reverse transcription. Virus Res.

[48] Santiago ML, Greene WC, Domingo E, Parrish CR, Holland JJ (2008). The role of the APOBEC3 family of cytidine deaminases in innate immunity, G-to-A hypermutation, and evolution of retroviruses. Origin and Evolution of Viruses.

[49] Harris RS, Bishop KN, Sheehy AM, Craig HM, Petersen-Mahrt SK, Watt IN, Neuberger MS, Malim MH (2003). DNA deamination mediates innate immunity to retroviral infection. Cell.

[50] Mangeat B, Turelli P, Caron G, Friedli M, Perrin L, Trono D (2003). Broad antiretroviral defence by human APOBEC3G through lethal editing of nascent reverse transcripts. Nature.

[51] Zhang H, Yang B, Pomerantz RJ, Zhang C, Arunachalam SC, Gao L (2003). The cytidine deaminase CEM15 induces hypermutation in newly synthesized HIV-1 DNA. Nature.

[52] Bishop KN, Holmes RK, Malim MH (2006). Antiviral potency of APOBEC proteins does not correlate with cytidine deamination. J Virol.

[53] Holmes RK, Koning FA, Bishop KN, Malim MH (2007). APOBEC3F can inhibit the accumulation of HIV-1 reverse transcription products in the absence of hypermutation. Comparisons with APOBEC3G. J Biol Chem.

[54] Bishop KN, Verma M, Kim E-Y, Wolinsky SM, Malim MH (2008). APOBEC3G inhibits elongation of HIV-1 reverse transcripts. PLoS Pathog.

[55] Turelli P, Mangeat B, Jost S, Vianin S, Trono D (2004). Inhibition of hepatitis B virus replication by APOBEC3G. Science.

[56] Chen H, Lilley CE, Yu Q, Lee DV, Chou J, Narvaiza I, Landau NR, Weitzman MD (2006). APOBEC3A is a potent inhibitor of adeno-associated virus and retrotransposons. Curr Biol.

[57] Okeoma CM, Lovsin N, Peterlin BM, Ross SR (2007). APOBEC3 inhibits mouse mammary tumour virus replication *in vivo*. Nature.

[58] Holmes RK, Malim MH, Bishop KN (2007). APOBEC-mediated viral restriction: not simply editing. Trends Biochem Sci.

[59] Zhang WH, Svarovskaia ES, Barr R, Pathak VK (2002). Y586F mutation in murine leukemia virus reverse transcriptase decreases fidelity of DNA synthesis in regions associated with adenine-thymine tracts. Proc Natl Acad Sci USA.

[60] Rascle J-B, Ficheux D, Darlix J-L (1998). Possible roles of nucleocapsid protein of MoMuLV in the specificity of proviral DNA synthesis and in the genetic variability of the virus. J Mol Biol.

[61] Post K, Kankia B, Gopalakrishnan S, Yang V, Cramer E, Saladores P, Gorelick RJ, Guo J, Musier-Forsyth K, Levin JG (2009). Fidelity of plus-strand primer requires the nucleic acid chaperone activity of HIV-1 nucleocapsid protein. Nucleic Acids Res.

[62] Bakhanashvili M (2001). p53 enhances the fidelity of DNA synthesis by human immunodeficiency virus type 1 reverse transcriptase. Oncogene.

[63] Bakhanashvili M, Novitsky E, Lilling G, Rahav G (2004). p53 in cytoplasm may enhance the accuracy of DNA synthesis by human immunodeficiency virus type 1 reverse transcriptase. Oncogene.

[64] Menéndez-Arias L (2002). Molecular basis of fidelity of DNA synthesis and nucleotide specificity of retroviral reverse transcriptases. Prog Nucleic Acid Res Mol Biol.

[65] Bebenek K, Kunkel TA (1995). Analyzing fidelity of DNA polymerases. Methods Enzymol.

[66] Ji J, Loeb LA (1994). Fidelity of HIV-1 reverse transcriptase copying a hypervariable region of the HIV-1 *env* gene. Virology.

[67] Ji J, Loeb LA (1992). Fidelity of HIV-1 reverse transcriptase copying RNA *in vitro*. Biochemistry.

[68] Boyer PL, Hughes SH (2000). Effects of amino acid substitutions at position 115 on the fidelity of human immunodeficiency virus type 1 reverse transcriptase. J Virol.

[69] Roberts JD, Bebenek K, Kunkel TA (1988). The accuracy of reverse transcriptase from HIV-1. Science.

[70] Bebenek K, Abbotts J, Roberts JD, Wilson SH, Kunkel TA (1989). Specificity and mechanism of error-prone replication by human immunodeficiency virus-1 reverse transcriptase. J Biol Chem.

[71] Boyer JC, Bebenek K, Kunkel TA (1992). Unequal human immunodeficiency virus type 1 reverse transcriptase error rates with RNA and DNA templates. Proc Natl Acad Sci USA.

[72] Eckert KA, Kunkel TA (1993). Fidelity of DNA synthesis catalyzed by human DNA polymerase α and HIV-1 reverse transcriptase: effect of reaction pH. Nucleic Acids Res.

[73] Drosopoulos WC, Prasad VR (1998). Increased misincorporation fidelity observed for nucleoside analog resistance mutations M184V and E89G in human immunodeficiency virus type 1 reverse transcriptase does not correlate with the overall error rate measured *in vitro*. J Virol.

[74] Rezende LF, Drosopoulos WC, Prasad VR (1998). The influence of 3TC resistance mutation M184I on the fidelity and error specificity of human immunodeficiency virus type 1 reverse transcriptase. Nucleic Acids Res.

[75] Fisher TS, Prasad VR (2002). Substitutions of Phe^61^ located in the vicinity of template 5′-overhang influence polymerase fidelity and nucleoside analog sensitivity of HIV-1 reverse transcriptase. J Biol Chem.

[76] Weiss KK, Chen R, Skasko M, Reynolds HM, Lee K, Bambara RA, Mansky LM, Kim B (2004). A role for dNTP binding of human immunodeficiency virus type 1 reverse transcriptase in viral mutagenesis. Biochemistry.

[77] Curr K, Tripathi S, Lennerstrand J, Larder BA, Prasad VR (2006). Influence of naturally occurring insertions in the fingers subdomain of human immunodeficiency virus type 1 reverse transcriptase on polymerase fidelity and mutation frequencies *in vitro*. J Gen Virol.

[78] Matamoros T, Kim B, Menéndez-Arias L (2008). Mechanistic insights into the role of Val75 of HIV-1 reverse transcriptase in misinsertion and mispair extension fidelity of DNA synthesis. J Mol Biol.

[79] Álvarez MM, Matamoros T, Menéndez-Arias L (2009). Increased thermostability and fidelity of DNA synthesis of wild-type and mutant HIV-1 group O reverse transcriptases. J Mol Biol.

[80] Boyer PL, Stenbak CR, Hoberman D, Linial ML, Hughes SH (2007). *In vitro* fidelity of the prototype primate foamy virus (PFV) RT compared to HIV-1 RT. Virology.

[81] Stuke AW, Ahmad-Omar O, Hoefer K, Hunsmann G, Jentsch KD (1997). Mutations in the SIV *env* and the M13 *lac*Zα gene generated *in vitro* by reverse transcriptases and DNA polymerases. Arch Virol.

[82] Diamond TL, Souroullas G, Weiss KK, Lee KY, Bambara RA, Dewhurst S, Kim B (2003). Mechanistic understanding of an altered fidelity simian immunodeficiency virus reverse transcriptase mutation V148I, identified in a pig-tailed macaque. J Biol Chem.

[83] Operario DJ, Reynolds HM, Kim B (2005). Comparison of DNA polymerase activities between recombinant feline immunodeficiency and leukema virus reverse transcriptases. Virology.

[84] Roberts JD, Preston BD, Johnston LA, Soni A, Loeb LA, Kunkel TA (1989). Fidelity of two retroviral reverse transcriptases during DNA-dependent DNA synthesis *in vitro*. Mol Cell Biol.

[85] Pathak VK, Temin HM (1990). Broad spectrum of *in vivo* forward mutations, hypermutations, and mutational hotspots in a retroviral shuttle vector after a single replication cycle: substitutions, frameshifts, and hypermutations. Proc Natl Acad Sci USA.

[86] Bebenek K, Abbotts J, Wilson SH, Kunkel TA (1993). Error-prone polymerization by HIV-1 reverse transcriptase – Contribution of template-primer misalignment, miscoding, and termination probability to mutational hot spots. J Biol Chem.

[87] Quiñones-Mateu ME, Soriano V, Domingo E, Menéndez-Arias L (1997). Characterization of the reverse transcriptase of a human immunodeficiency virus type 1 group O isolate. Virology.

[88] Menéndez-Arias L, Abraha A, Quiñones-Mateu ME, Mas A, Camarasa M-J, Arts EJ (2001). Functional characterization of chimeric reverse transcriptases with polypeptide subunits of highly divergent HIV-1 group M and O strains. J Biol Chem.

[89] Gärtner K, Wiktorowicz T, Park J, Mergia A, Rethwilm A, Scheller C (2009). Accuracy estimation of foamy virus genome copying. Retrovirology.

[90] Hübner A, Kruhoffer M, Grosse F, Krauss G (1992). Fidelity of human immunodeficiency virus type I reverse transcriptase in copying natural RNA. J Mol Biol.

[91] Kim T, Mudry RA, Rexrode CA, Pathak VK (1996). Retroviral mutation rates and A-to-G hypermutations during different stages of retroviral replication. J Virol.

[92] Echols H, Goodman MF (1991). Fidelity mechanisms in DNA replication. Annu Rev Biochem.

[93] Johnson KA (1993). Conformational coupling in DNA polymerase fidelity. Annu Rev Biochem.

[94] Kati WM, Johnson KA, Jerva LF Jerva, Anderson KS (1992). Mechanism and fidelity of HIV reverse transcriptase. J Biol Chem.

[95] Zinnen S, Hsieh J-C, Modrich P (1994). Misincorporation and mispaired primer extension by human immunodeficiency virus reverse transcriptase. J Biol Chem.

[96] Kerr SG, Anderson KS (1997). RNA dependent DNA replication fidelity of HIV-1 reverse transcriptase: Evidence of discrimination between DNA and RNA substrates. Biochemistry.

[97] Weiss KK, Bambara RA, Kim B (2002). Mechanistic role of residue Gln^151^ in error prone DNA synthesis by human immunodeficiency virus type 1 (HIV-1) reverse transcriptase (RT) – Pre-steady state kinetic study of the Q151N HIV-1 RT mutant with increased fidelity. J Biol Chem.

[98] Skasko M, Weiss KK, Reynolds HM, Jamburuthugoda V, Lee K, Kim B (2005). Mechanistic differences in RNA-dependent DNA polymerization and fidelity between murine leukemia virus and HIV-1 reverse transcriptases. J Biol Chem.

[99] Perrino FW, Preston BD, Sandell LL, Loeb LA (1989). Extension of mismatched 3′ termini of DNA is a major determinant of the infidelity of human immunodeficiency virus type 1 reverse transcriptase. Proc Natl Acad Sci USA.

[100] Yu H, Goodman MF (1992). Comparison of HIV-1 and avian myeloblastosis virus reverse transcriptase fidelity on RNA and DNA templates. J Biol Chem.

[101] Bakhanashvili M, Hizi A (1992). Fidelity of the reverse transcriptase of human immunodeficiency virus type 2. FEBS Lett.

[102] Bakhanashvili M, Hizi A (1992). Fidelity of the RNA-dependent DNA synthesis exhibited by the reverse transcriptases of human immunodeficiency virus types 1 and 2 and of murine leukemia virus: Mispair extension frequencies. Biochemistry.

[103] Bakhanashvili M, Hizi A (1993). Fidelity of DNA synthesis exhibited *in vitro* by the reverse transcriptase of the lentivirus equine infectious anemia virus. Biochemistry.

[104] Avidan O, Bochner R, Hizi A (2006). The catalytic properties of the recombinant reverse transcriptase of bovine immunodeficiency virus. Virology.

[105] Avidan O, Meer ME, Oz I, Hizi A (2002). The processivity and fidelity of DNA synthesis exhibited by the reverse transcriptase of bovine leukemia virus. Eur J Biochem.

[106] Taube R, Avidan O, Bakhanashvili M, Hizi A (1998). DNA synthesis exhibited by the reverse transcriptase of mouse mammary tumor virus: Processivity and fidelity of misinsertion and mispair extension. Eur J Biochem.

[107] Avidan O, Loya S, Tönjes RR, Sevilya Z, Hizi A (2003). Expression and characterization of a recombinant novel reverse transcriptase of a porcine endogenous retrovirus. Virology.

[108] Boutabout M, Wilhelm M, Wilhelm F-X (2001). DNA synthesis fidelity by the reverse transcriptase of the yeast retrotransposon Ty1. Nucleic Acids Res.

[109] Conlan LH, Stanger MJ, Ichiyanagi K, Belfort M (2005). Localization, mobility and fidelity of retrotransposed group II introns in rRNA genes. Nucleic Acids Res.

[110] Kirshenboim N, Hayouka Z, Friedler A, Hizi A (2007). Expression and characterization of a novel reverse transcriptase of the LTR retrotransposon Tf1. Virology.

[111] Menéndez-Arias L (2008). Mechanisms of resistance to nucleoside analogue inhibitors of HIV-1 reverse transcriptase. Virus Res.

[112] Sarafianos SG, Marchand B, Das K, Himmel DM, Parniak MA, Hughes SH, Arnold E (2009). Structure and function of HIV-1 reverse transcriptase: molecular mechanisms of polymerization and inhibition. J Mol Biol.

[113] Kohlstaedt LA, Wang J, Friedman JM, Rice PA, Steitz TA (1992). Crystal structure at 3.5 Å resolution of HIV-1 reverse transcriptase complexed with an inhibitor. Science.

[114] Huang H, Chopra R, Verdine GL, Harrison SC (1998). Structure of a covalently trapped catalytic complex of HIV-1 reverse transcriptase: Implications for drug resistance. Science.

[115] Mendieta J, Cases-González CE, Matamoros T, Ramírez G, Menéndez-Arias L (2008). A Mg^2+^-induced conformational switch rendering a competent DNA polymerase catalytic complex. Proteins.

[116] Steitz TA, Smerdon SJ, Jäger J, Joyce CM (1994). A unified polymerase mechanism for nonhomologous DNA and RNA polymerases. Science.

[117] Shah FS, Curr KA, Hamburgh ME, Parniak M, Mitsuya H, Arnez JG, Prasad VR (2000). Differential influence of nucleoside analog-resistance mutations K65R and L74V on the overall mutation rate and error specificity of human immunodeficiency virus type 1 reverse transcriptase. J Biol Chem.

[118] Lewis DA, Bebenek K, Beard WA, Wilson SH, Kunkel TA (1999). Uniquely altered DNA replication fidelity conferred by an amino acid change in the nucleotide binding pocket of human immunodeficiency virus type 1 reverse transcriptase. J Biol Chem.

[119] Jonckheere H, De Clercq E, Anné J (2000). Fidelity analysis of HIV-1 reverse transcriptase mutants with an altered amino-acid sequence at residues Leu74, Glu89, Tyr115, Tyr183 and Met184. Eur J Biochem.

[120] Kim B, Hathaway TR, Loeb LA (1998). Fidelity of mutant HIV-1 reverse transcriptases: Interaction with the single-stranded template influences the accuracy of DNA synthesis. Biochemistry.

[121] Kim B, Ayran JC, Sagar SG, Adman ET, Fuller SM, Tran NH, Horrigan J (1999). New human immunodeficiency virus, type 1 reverse transcriptase (HIV-1 RT) mutants with increased fidelity of DNA synthesis – Accuracy, template binding, and processivity. J Biol Chem.

[122] Hamburgh ME, Curr KA, Monaghan M, Rao VR, Tripathi S, Preston BD, Sarafianos S, Arnold E, Darden T, Prasad VR (2006). Structural determinants of slippage-mediated mutations by human immunodeficiency virus type 1 reverse transcriptase. J Biol Chem.

[123] Rezende LF, Curr K, Ueno T, Mitsuya H, Prasad VR (1998). The impact of multidideoxynucleoside resistance-conferring mutations in human immunodeficiency virus type 1 reverse transcriptase on polymerase fidelity and error specificity. J Virol.

[124] Weiss KK, Isaacs SJ, Tran NH, Adman ET, Kim B (2000). Molecular architecture of the mutagenic active site of human immunodeficiency virus type 1 reverse transcriptase: roles of the β8-αE loop in fidelity, processivity, and substrate interactions. Biochemistry.

[125] Beard WA, Stahl SJ, Kim H-R, Bebenek K, Kumar A, Strub M-P, Becerra SP, Kunkel TA, Wilson SH (1994). Structure/function studies of human immunodeficiency virus type 1 reverse transcriptase – Alanine scanning mutagenesis of an α-helix in the thumb subdomain. J Biol Chem.

[126] Bebenek K, Beard WA, Casas-Finet JR, Kim H-R, Darden TA, Wilson SH, Kunkel TA (1995). Reduced frameshift fidelity and processivity of HIV-1 reverse transcriptase mutants containing alanine substitutions in helix H of the thumb subdomain. J Biol Chem.

[127] Beard WA, Minnick DT, Wade CL, Prasad R, Won RL, Kumar A, Kunkel TA, Wilson SH (1996). Role of the “helix clamp” in HIV-1 reverse transcriptase catalytic cycling as revealed by alanine-scanning mutagenesis. J Biol Chem.

[128] Gao G, Orlova M, Georgiadis MM, Hendrickson WA, Goff SP (1997). Conferring RNA polymerase activity to a DNA polymerase: A single residue in reverse transcriptase controls substrate selection. Proc Natl Acad Sci USA.

[129] Cases-González CE, Gutiérrez-Rivas M, Menéndez-Arias L (2000). Coupling ribose selection to fidelity of DNA synthesis: The role of Tyr-115 of human immunodeficiency virus type 1 reverse transcriptase. J Biol Chem.

[130] Martín-Hernández AM, Domingo E, Menéndez-Arias L (1996). Human immunodeficiency virus type 1 reverse transcriptase: role of Tyr115 in deoxynucleotide binding and misinsertion fidelity of DNA synthesis. EMBO J.

[131] Boyer PL, Sarafianos SG, Arnold E, Hughes SH (2000). Analysis of mutations at positions 115 and 116 in the dNTP binding site of HIV-1 reverse transcriptase. Proc Natl Acad Sci USA.

[132] Martín-Hernández AM, Gutiérrez-Rivas M, Domingo E, Menéndez-Arias L (1997). Mispair extension fidelity of human immunodeficiency virus type 1 reverse transcriptases with amino acid substitutions affecting Tyr115. Nucleic Acids Res.

[133] Boyer PL, Hughes SH (2000). Effects of amino acid substitutions at position 115 on the fidelity of human immunodeficiency virus type 1 reverse transcriptase. J Virol.

[134] Halvas EK, Svarovskaia ES, Pathak VK (2000). Role of murine leukemia virus reverse transcriptase deoxyribonucleoside triphosphate-binding site in retroviral replication and *in vivo* fidelity. J Virol.

[135] Olivares I, Sánchez-Merino V, Martínez MA, Domingo E, López-Galíndez C, Menéndez-Arias L (1999). Second-site reversion of a human immunodeficiency virus type 1 reverse transcriptase mutant that restores enzyme function and replication capacity. J Virol.

[136] Gutiérrez-Rivas M, Ibáñez A, Martínez MA, Domingo E, Menéndez-Arias L (1999). Mutational analysis of Phe-160 within the ‘palm’ subdomain of human immunodeficiency virus type 1 reverse transcriptase. J Mol Biol.

[137] Cases-González CE, Menéndez-Arias L (2005). Nucleotide specificity of HIV-1 reverse transcriptases with amino acid substitutions affecting Ala-114. Biochem J.

[138] Kaushik N, Talele TT, Pandey PK, Harris D, Yadav PNS, Pandey VN (2000). Role of glutamine 151 of human immunodeficiency virus type-1 reverse transcriptase in substrate selection as assessed by site-directed mutagenesis. Biochemistry.

[139] Wainberg MA, Drosopoulos WC, Salomon H, Hsu M, Borkow G, Parniak MA, Gu Z, Song Q, Manne J, Islam S, Castriota G, Prasad VR (1996). Enhanced fidelity of 3TC-selected mutant HIV-1 reverse transcriptase. Science.

[140] Hsu M, Inouye P, Rezende L, Richard N, Li Z, Prasad VR, Wainberg MA (1997). Higher fidelity of RNA-dependent DNA mispair extension by M184V drug-resistant than wild-type reverse transcriptase of human immunodeficiency virus type 1. Nucleic Acids Res.

[141] Oude Essink BB, Back NKT, Berkhout B (1997). Increased polymerase fidelity of the 3TC-resistant variants of HIV-1 reverse transcriptase. Nucleic Acids Res.

[142] Hamburgh ME, Drosopoulos WC, Prasad VR (1998). The influence of 3TC-resistance mutations E89G and M184V in the human immunodeficiency virus reverse transcriptase on mispair extension efficiency. Nucleic Acids Res.

[143] Sarafianos SG, Pandey VN, Kaushik N, Modak MJ (1995). Site-directed mutagenesis of arginine 72 of HIV-1 reverse transcriptase – Catalytic role and inhibitor sensitivity. J Biol Chem.

[144] Chowdhury K, Kaushik N, Pandey VN, Modak MJ (1996). Elucidation of the role of Arg 110 of murine leukemia virus reverse transcriptase in the catalytic mechanism: Biochemical characterization of its mutant enzymes. Biochemistry.

[145] Agopian A, Depollier J, Lionne C, Divita G (2007). p66 Trp24 and Phe61 are essential for accurate association of HIV-1 reverse transcriptase with primer/template. J Mol Biol.

[146] Fisher TS, Darden T, Prasad VR (2003). Substitutions at Phe61 in the β3–β4 hairpin of HIV-1 reverse transcriptase reveal a role for the fingers subdomain in strand displacement DNA synthesis. J Mol Biol.

[147] Mandal D, Dash C, Le Grice SFJ, Prasad VR (2006). Analysis of HIV-1 replication block due to substitutions at F61 residue of reverse transcriptase reveals additional defects involving the RNase H function. Nucleic Acids Res.

[148] Wisniewski M, Palaniappan C, Fu Z, Le Grice SFJ, Fay P, Bambara RA (1999). Mutations in the primer grip region of HIV reverse transcriptase can increase replication fidelity. J Biol Chem.

[149] Gutiérrez-Rivas M, Menéndez-Arias L (2001). A mutation in the primer grip region of HIV-1 reverse transcriptase that confers reduced fidelity of DNA synthesis. Nucleic Acids Res.

[150] Cases-González CE, Menéndez-Arias L (2004). Increased G→A transition frequencies displayed by primer grip mutants of human immunodeficiency virus type 1 reverse transcriptase. J Virol.

[151] Ding J, Das K, Hsiou Y, Sarafianos SG, Clark AD, Jacobo-Molina A, Tantillo C, Hughes SH, Arnold E (1998). Structure and functional implications of the polymerase active site region in a complex of HIV-1 RT with a double-stranded DNA template-primer and an antibody Fab fragment at 2.8 Å resolution. J Mol Biol.

[152] Sarafianos SG, Das K, Tantillo C, Clark AD, Ding J, Whitcomb JM, Boyer PL, Hughes SH, Arnold E (2001). Crystal structure of HIV-1 reverse transcriptase in complex with a polypurine tract RNA:DNA. EMBO J.

[153] McWilliams MJ, Julias JG, Sarafianos SG, Alvord WG, Arnold E, Hughes SH (2006). Combining mutations in HIV-1 reverse transcriptase with mutations in the HIV-1 polypurine tract affects RNase H cleavages involved in PPT utilization. Virology.

[154] Mbisa JL, Nikolenko GN, Pathak VK (2005). Mutations in the RNase H primer grip domain of murine leukemia virus reverse transcriptase decrease efficiency and accuracy of plus-strand DNA transfer. J Virol.

[155] Jonckheere H, Witvrouw M, De Clercq E, Anné J (1998). Lamivudine resistance of HIV type 1 does not delay development of resistance to nonnucleoside HIV type 1-specific reverse transcriptase inhibitors as compared with wild-type HIV type 1. AIDS Res Hum Retroviruses.

[156] Keulen W, van Wijk A, Schuurman R, Berkhout B, Boucher CAB (1999). Increased polymerase fidelity of lamivudine-resistant HIV-1 variants does not limit their evolutionary potential. AIDS.

[157] Diallo K, Brenner B, Oliveira M, Moisi D, Detorio M, Götte M, Wainberg MA (2003). The M184V substitution in human immunodeficiency virus type 1 reverse transcriptase delays the development of resistance to amprenavir and efavirenz in subtype B and C clinical isolates. Antimicrob Agents Chemother.

[158] Preston BD (1997). Reverse transcriptase fidelity and HIV-1 variation. Science.

[159] Loeb LA, Essigmann JM, Kazazi F, Zhang J, Rose KD, Mullins JI (1999). Lethal mutagenesis of HIV with mutagenic nucleoside analogs. Proc Natl Acad Sci USA.

[160] Loeb LA, Mullins JI (2000). Lethal mutagenesis of HIV by mutagenic ribonucleoside analogs. AIDS Res Hum Retroviruses.

[161] Domingo E, Escarmís C, Lázaro E, Manrubia SC (2005). Quasispecies dynamics and RNA virus extinction. Virus Res.

[162] Pathak VK, Temin HM (1992). 5-Azacytidine and RNA secondary structure increase the retrovirus mutation rate. J Virol.

[163] Crotty S, Cameron CE, Andino R (2001). RNA virus error catastrophe: direct molecular test by using ribavirin. Proc Natl Acad Sci USA.

[164] Harris KS, Brabant W, Styrchak S, Gall A, Daifuku R (2005). KP-1212/1461, a nucleoside designed for the treatment of HIV by viral mutagenesis. Antiviral Res.

[165] Graci JD, Harki DA, Korneeva VS, Edathil JP, Too K, Franco D, Smidansky ED, Paul AV, Peterson BR, Brown DM, Loakes D, Cameron CE (2007). Lethal mutagenesis of poliovirus mediated by a mutagenic pyrimidine analogue. J Virol.

[166] LaCasse RA, Remington KM, North TW (1996). The mutation frequency of feline immunodeficiency virus enhanced by 3′-azido-3′-deoxythymidine. J Acquir Immune Defic Syndr Hum Retrovirol.

[167] Julias JG, Kim T, Arnold G, Pathak VK (1997). The antiretrovirus drug 3′-azido-3′-deoxythymidine increases the retrovirus mutation rate. J Virol.

[168] Mansky LM, Bernard LC (2000). 3′-azido-3′-deoxythymidine (AZT) and AZT-resistant reverse transcriptase can increase the *in vivo* mutation rate of human immunodeficiency virus type 1. J Virol.

[169] Dapp MJ, Clouser CL, Patterson S, Mansky LM (2009). 5-Azacytidine can induce lethal muta-genesis in human immunodeficiency virus type 1. J Virol.

[170] Julias JG, Pathak VK (1998). Deoxyribonucleoside triphosphate pool imbalances *in vivo* are associated with an increased retroviral mutation rate. J Virol.

[171] Balzarini J, Camarasa M-J, Pérez-Pérez M-J, San-Félix A, Velázquez S, Perno C-F, De Clercq E, Anderson JN, Karlsson A (2001). Exploitation of the low fidelity of human immunodeficiency virus type 1 (HIV-1) reverse transcriptase and the nucleotide composition bias in the HIV-1 genome to alter the drug resistance development of HIV. J Virol.

